# Levodopa exacerbated tremor in Parkinson's disease

**DOI:** 10.3389/fneur.2026.1866044

**Published:** 2026-08-12

**Authors:** José Fidel Baizabal-Carvallo, Marlene Alonso-Juarez, Robert Fekete

**Affiliations:** 1 Department of Sciences and Engineering, University of Guanajuato, León, Mexico; 2 Instituto Politécnico Nacional, Mexico City, Mexico; 3 Department of Neurology, New York Medical College, Valhalla, NY, United States

**Keywords:** cerebellum, dyskinesia, levodopa, Parkinson's disease, tremor

## Abstract

**Background:**

Levodopa is considered the most effective pharmacological therapy for Parkinson's disease (PD). However, it can cause side effects including diverse types of dyskinesia. In rare cases, tremor worsening has been reported in patients with PD taking levodopa. The frequency of this effect in clinical practice is unclear, and the phenomenology is poorly characterized.

**Objectives:**

We aimed to assess the frequency, clinical features, and factors associated with tremor exacerbated by levodopa in patients with PD.

**Methods:**

We studied a cohort of 274 consecutive patients with PD and determined the frequency of tremor exacerbated by levodopa.

**Results:**

Tremor exacerbation with levodopa was identified in four patients (1.46%) and 1.55% of patients taking levodopa. There were two men and two women, with ages ranging from 53 to 73 years at the time of evaluation. All patients experienced a consistent worsening of rest tremor within 20 min of taking a low dose of levodopa (62.5 mg or 125 mg). No benefit from biperiden or amantadine was observed for levodopa-exacerbated tremor in any of the cases. None of the patients showed signs of atypical parkinsonism or abnormal MRI. Patients with levodopa-exacerbated tremor were very reluctant to take more than a small dose of levodopa or any dose at all, hampering potential benefits for other parkinsonian features such as rigidity, bradykinesia, and parkinsonian gait.

**Conclusion:**

Tremor exacerbated by levodopa is an uncommon side effect in patients with PD that limits the benefit of this therapy.

## Introduction

Parkinson's disease (PD) is a neurodegenerative disorder characterized by loss of dopaminergic cells in the substantia nigra (1). PD presents with several motor and non-motor manifestations. Among motor symptoms, tremor is one of the most common and disabling manifestations (2). Tremors in PD can occur in the craniocervical region, but they more commonly affect the upper and lower limbs (3, 4). While rest tremor is observed in approximately 70% of patients with PD, postural and kinetic tremors are not uncommon and are identified in up to 50% of patients (5–8). Rest tremor may be observed alone or combined with postural and kinetic tremor in patients with PD (9).

Treatment of motor symptoms in PD, including tremors, is based on dopaminergic medications, where levodopa is considered the most effective drug (1). Levodopa therapy is limited by several side effects, including nausea, vomiting, drowsiness, and hypotension. However, dyskinesia is the most feared levodopa-induced side effect in patients with PD and may present as chorea, dystonia, ballism, myoclonus, or a combination of these abnormal movements (10). Dyskinesia is typically classified based on when it occurs during the levodopa dosing cycle. In addition to dyskinesia, some patients with PD experience tremor exacerbation following a dose of levodopa (11). Spectral analysis of sustained hand force showed worsening tremor and dyskinesia following a single dose of levodopa in six patients with PD who were assessed in a controlled environment (12). Despite this evidence, the frequency of levodopa-exacerbated tremor in PD remains uncertain in clinical settings, along with its clinical features and correlations; therefore, we aimed to assess these features in a cohort of patients with PD.

## Materials and methods

We reviewed the clinical response to dopaminergic medications in consecutive patients with PD attending a tertiary-care center for movement disorders. All patients were evaluated by a movement disorders specialist (JFB-C), who has over 15 years of experience in treating PD. The diagnosis of PD was based on the Queen Square Brain Bank Criteria (13). Patients were classified according to the clinical presentation into three categories: (1) tremor-dominant, when tremor was the predominant motor feature; (2) rigid-akinetic, when muscle rigidity and bradykinesia dominated the clinical presentation; and (3) mixed, when tremor, rigidity, and/or bradykinesia were all present without a dominant clinical feature.

The severity of parkinsonism was evaluated using the Movement Disorders Society Unified Parkinson's Disease Rating Scale part III (MDS-UPDRS-III) or motor score (14). The disease stage was determined according to the modified Hoehn & Yahr scale (15). The cognitive evaluation was performed using the Montreal Cognitive Assessment (MoCA). We assessed the type of tremor, body distribution, amplitude, and response to therapy in each case. Patients or their family members who reported tremor exacerbations with levodopa were identified during the initial interview and clinical examination and were subsequently reevaluated to confirm this phenomenon. Tremor exacerbation was defined as an increase of at least one point in the tremor items from the MDS-UPDRS-III, occurring 15–20 min after a standard dose of 125 mg of levodopa **(**half a tablet of levodopa/carbidopa, 250/25 mg). Patients experiencing levodopa-exacerbated tremor were asked to present in the “Off” medication state for examination. After this evaluation, they received a standard dose of levodopa and were reevaluated for tremor exacerbation within the next 2–3 h in the medication “On” state. In two cases, tremor frequency was measured using the “Tremor Analysis” application. We evaluated whether levodopa-exacerbated tremor improved with the anticholinergic biperiden and amantadine. Patients identified with levodopa-exacerbated tremor provided written informed consent for video and photography and to participate in the study under an anonymized identity. The study was approved by the local research and ethics committee and was conducted in accordance with the Declaration of Helsinki.

## Results

We studied 274 consecutive patients with PD (153 men and 121 women), with a mean age (SD) at presentation of 67.92 ± 10.70 years (ranging from 33 to 90 years); 94% were receiving treatment with levodopa. Tremor worsening associated with levodopa was identified in four patients, which represents 1.46% of the whole cohort (1.55% among those receiving levodopa). The affected individuals included two men and two women, aged 73, 61, 60, and 53 years at the time of the evaluation. Two patients were classified as tremor-dominant and two patients as having mixed PD. The clinical and demographic features of these patients are summarized in Table 1. No patient had moderate or severe autonomic dysfunction, hallucinations, vertical gaze palsy, or any other sign suggesting atypical parkinsonism. No severe cognitive decline was observed in any of these four patients, with MoCA scores ranging from 21 to 29 points. Brain MRI scans were unremarkable in all cases. There was no family history of PD in cases 1–3; however, case 4 reported a possible history of PD in her father.

**Table 1:** Summary of clinical features of patients with Parkinson's disease and levodopa-induced tremor.

Feature	Case 1	Case 2	Case 3	Case 4
Age at onset (years)/sex	73/M	57/M	42/F	49/F
Age at evaluation (years)	73	60	53	61
Type of PD	Tremor-dominant	Mixed	Mixed	Tremor-dominant
Modified Hoehn–Yahr stage at evaluation	3	3	3	3
MDS-UPDRS-III score at first evaluation	12 “Off”	49 “Off”	90 “Off”	33 “Off”
MoCA	27	24	21	29
Time since PD onset and the first trial of levodopa	3 months	3 years	5 years	2 years
Smallest levodopa dose identified to exacerbate tremor	125 mg	125 mg	125 mg	62.5 mg
Family history of PD	Absent	Absent	Absent	Father with probable PD
Neuroimaging (MRI)	Normal	Normal	Normal	Normal
Rest tremor in levodopa “Off” state	Bilateral upper limb rest tremor 2+ and lower limbs 1+, persistence 3+ (4.7 Hz)	Bilateral upper limb rest tremor 1+ and lower limbs 1+, persistence 3+ (4.3 Hz)	Bilateral upper limb rest tremor 2+ and lower limbs 3+, persistence 3+	Left upper limb rest tremor 2+ and left lower limb 1+, persistence 4+
Rest tremor in levodopa “On” state	Tremor exacerbation in the upper and lower limbs	Mild tremor exacerbation, persistent in four limbs with mild trunk tremor	Tremor exacerbated mostly in the lower limbs but persistent in the upper limbs	Tremor appears on both sides during exacerbation
Action tremor: postural (P) and kinetic (k)	Left side k1+	None or very subtle	None, but appearing with levodopa: p2+, k 2+ bilateral	Left side p1+, k2+ tremor, some exacerbation by levodopa
Dyskinesia (choreic, dystonic)	Yes, left lower limb painful dystonia in the “On” state	None	Left lower limb dystonia in the “Off” state	Mild “On” choreic axial and limb dyskinesia
Tremor induced by a dopaminergic agonist	Unknown	None	Probably (Pramipexole)	None
Tremor exacerbation, response to other medications	No response to biperiden or amantadine (improvement in gait)	No response to amantadine or biperiden	No response to amantadine or biperiden	No response to pramipexole, amantadine, and biperiden
Current treatment	Levodopa 62.5 mg TID, amantadine 100 mg BID, rasagiline 1 mg/day	Rasagiline 1 mg/day, levodopa 125 mg at bedtime to help sleep.	Levodopa 62.5 mg TID	Pramipexole 1 mg TID, amantadine 100 mg BID, biperiden 2 mg TID

F, female; k, kinetic; M, male; MoCA, Montreal Cognitive Assessment; MRI, magnetic resonance imaging; PD, Parkinson's disease; p, postural.

All patients reported tremor exacerbation within 20 min of taking levodopa (combined with carbidopa in all cases). The effect was noticed even with low doses of immediate-release levodopa: one patient received 62.5 mg per dose ($\frac{1}{4}$ of levodopa/carbidopa 250/25 mg tablet), while three patients received 125 mg per dose (1/2 a tablet of levodopa/carbidopa 250/25 mg). Tremor was reported in all cases during the medication “On” state. Case 4 showed mild axial and limb dyskinesia in the medication “On” state, and cases 1 and 2 had painful dystonia in the “Off” state.

The exacerbated tremor was perceived as generalized, low-amplitude, and very uncomfortable in all cases. Patients markedly refused to take more than a low dose of levodopa or any dose at all, despite experiencing certain benefits in rigidity, bradykinesia, and gait. We confirmed that this phenomenology was not levodopa-induced dyskinesia (see Supplementary video). We tried to improve levodopa-exacerbated tremor with biperiden and amantadine, but no benefit was observed in any case. It is unclear whether dopamine agonists also exacerbated tremor in these patients, although case 3 reported increased tremor amplitud with low doses of pramipexole; case 4 took pramipexole, but she did not report tremor worsening with this medication. Deep brain stimulation (DBS) therapy was offered, but it was declined in all instances.

Cases 1–3 opted to take only a very low dose of immediate-release levodopa/carbidopa to avoid tremor exacerbation, even though this decision resulted in incomplete improvement of other parkinsonian symptoms. In contrast, case 4 strongly refused to take any dose of levodopa.

## Discussion

In this study, we present four cases of PD in which tremors were exacerbated by levodopa. All patients had otherwise typical PD without severe cognitive decline or signs of atypical parkinsonism, with a lack of abnormal neuroimaging or family history of neurodegeneration. Tremor exacerbated by levodopa (or dopaminergic agonists) was uncommon in our cohort, observed in 1.46% of patients with PD and 1.55% of those taking levodopa. This figure clearly contrasts with the study by Caligiuri and Lohr, which found that postural tremor occurred in six out of eight male patients with PD after a single dose of levodopa. The mean age of these patients was 71.8 years, and they also experienced levodopa peak-dose dyskinesia (12). In that study, tremor was observed within 45 min of taking levodopa, and its maximal amplitude usually coincided with the maximal amplitude of dyskinesia, except for two patients where the peak in tremor amplitude preceded dyskinesia (12). A similar correlation between peak tremor amplitude and dyskinesia following exposure to levodopa was also reported by Yanagisawa and Nezu a few years earlier (11).

Tremors exacerbated by levodopa should be distinguished from other possibilities. One is that patients may be receiving suboptimal doses of levodopa with tremor persisting in the “On” state. However, when tremor is exacerbated by levodopa, patients perceive an increase in tremor amplitude and even tend to refuse taking larger doses; this does not occur with levodopa underdosing. Another possibility is that our patients had dopamine-resistant rest tremors. In a study of 76 tremulous patients with PD, a challenge dose of levodopa was offered, and tremor severity was quantified by accelerometry (16). A proportion of patients was identified as having dopamine-resistant rest tremor (16). However, in a retrospective study involving 526 patients with PD who were evaluated for DBS, only 4.0% were categorized as non-responsive to levodopa and defined as tremor improvement of less than 25% (17). This percentage was lower than for bradykinesia (19.4%) and rigidity (9.8%); in that study, tremor was the most responsive motor feature, which was documented in 86.6% of cases (17). Moreover, patients with non-responsive rest tremor showed lower gastrointestinal and cardiovascular manifestations with a lower prevalence of REM-sleep behavior disorder and higher dopaminergic asymmetry compared with therapy-responsive patients, suggesting a distinct clinical/pathophysiological phenotype (18). In our study, patients showed tremor exacerbation rather than being “tremor-resistant.” As a result, they were reluctant to take more than a small dose of levodopa or any dose at all.

Dyskinesia during the “On” state is frequently confused with tremor in patients with PD. It is, therefore, of paramount importance to clarify which phenomenology occurs in these patients. In our study, a single patient showed “On” dystonic dyskinesia associated with tremor (case 1), and one patient showed mild choreic dyskinesia in the medication “On” state (case 4); however, two cases did not show dyskinesia during levodopa-induced tremor exacerbation, supporting the notion that the two phenomena may not always coincide. More straightforwardly, it is unclear why tremor exacerbated by levodopa is rarely observed in clinical practice outside a research laboratory; accordingly, this phenomenon may not be uncommon but may go undetected in certain cases, owing to its low amplitude or short duration. Tremor exacerbated by levodopa should not be confused with the wearing-off phenomenon, where tremor may reemerge at the end of the levodopa cycle. In our patients, tremor exacerbation was perceived within the first few minutes following levodopa intake, ruling out motor fluctuations misinterpreted as symptom exacerbation.

The pathophysiology of tremor in PD is complex and attributed to abnormal high-power oscillations in the cortical-basal ganglia and cerebello-thalamo-cortical circuits (19). An interaction of these circuits has been proposed in the “dimmer-switch model,” where the basal ganglia trigger tremor episodes, and the cerebello-thalamo-cortical circuit controls its amplitude (19). More recent evidence using functional MRI has shown that PD patients with levodopa-resistant tremor have increased tremor-related activity in non-dopaminergic areas such as the cerebellum, whereas patients with levodopa-responsive tremor show increased tremor-related activity in the thalamus and the secondary somatosensory area (20).

A shift from the parkinsonian beta frequency (15–30 Hz) to lower oscillatory frequencies in the theta (4–7 Hz) and alpha (8–12 Hz) ranges has been recorded in the globus pallidus interna (GPi) (21, 22) and the subthalamic nucleus (STN) (23, 24) of patients with PD following levodopa administration or DBS. This change has coincided with a marked improvement in parkinsonian symptoms (25). Patients with tremor-dominant PD show oscillatory activity in the subthalamic nucleus (STN) at the individual tremor frequency (or at double that frequency) between 4 and 8 Hz, with the onset recorded 150 milliseconds to several seconds after the tremor onset (26, 27). Studies assessing local field potentials in the STN of patients with PD and dyskinesia have shown a 77.6% increase in the 4–10 Hz band following a single dose of the dopamine agonist apomorphine or of levodopa, with a further increase of 17.8% in the gamma band (60–80 Hz) (28). Accordingly, it can be hypothesized that in patients with tremor exacerbated by levodopa, levodopa induces an abnormal shift in oscillatory activity within the STN and GPi toward theta or low alpha frequencies, which may eventually trigger tremor. Moreover, shifting abnormal oscillatory beta activity within the basal ganglia to the alpha and gamma bands following levodopa administration may cause a coincidental association between peak-dose choreic dyskinesia and tremor exacerbated by levodopa, as observed in case 4.

According to the dimmer-switch model, it is possible that such tremor may be perpetuated by cerebello-thalamic-cortical pathways under unclear mechanisms, making it resistant to the antitremorigenic effects of anticholinergics, weak dopaminergics, or N-methyl-D-aspartate non-competitive antagonists such as amantadine, as observed in our patients. Another intriguing question is why tremor exacerbation by levodopa was uncommonly observed in our patients and rarely reported in the medical literature. An intrinsic vulnerability to “dyskinetic/tremorigenic” oscillatory activity in the basal ganglia may exist in certain patients with PD that renders them prone to develop tremor exacerbation with levodopa.

A second dopaminergic hypothesis is related to the preservation of certain dopaminergic pathways. There is neuroimaging evidence that relative sparing of dopaminergic terminals in the caudate nucleus is a feature of rest tremor in PD (29). It is possible that levodopa-induced suprathreshold dopaminergic stimulation of the caudate-thalamic circuits in these patients paradoxically amplifies their tremor oscillations, but this hypothesis would also require experimental confirmation.

Another hypothesis may relate to adrenergic activation following levodopa intake. In this instance, increased levels of noradrenaline (NA) in the central nervous system may enhance the amplitude of parkinsonian tremor. This phenomenon has been confirmed experimentally by the administration of intravenous adrenaline in patients with PD (30). Indeed, evidence suggests that interneurons in the noradrenergic locus coeruleus of patients with tremor-dominant PD show less degeneration and a higher density of NA receptors compared with patients with other PD phenotypes and normal controls (31, 32). However, in our patients, we did not notice any signs of sympathetic hyperactivity related to tremor exacerbation with levodopa, such as an accelerated heart rate (≥10 beats/minute) or an increase in blood pressure levels (≥20 mmHg systolic/≥10 mmHg diastolic).

Serotoninergic neurons are also implicated in neurodegeneration in PD. Neuroimaging studies using 123ioflupane-fluoropropyl-carbomethoxy-3-beta-4-iodophenyltropane (123I-FP-CIT) single-photon emission computed tomography (SPECT) have shown an inverse correlation between raphe serotonin transporter availability and rest tremor severity in patients with tremor-dominant PD (7). This was also observed with raphe/putamen ratios, where more severe tremor scores were correlated with lower ratios. Moreover, the percentage of improvement in rest tremor amplitude after dopaminergic therapy was smaller in patients with lower raphe/putamen uptake ratio values, suggesting the possibility that serotoninergic dysfunction may contribute to tremor exacerbated by levodopa (7). Although the hypotheses for tremor exacerbation with levodopa are not mutually exclusive and more than one may potentially contribute to this phenomenon in a single patient, we favor the shifting basal ganglia oscillatory hypothesis due to accumulated experimental evidence.

The first step in treatment is to determine whether the patient has levodopa-exacerbated tremor, as this phenomenon should be clearly differentiated from the “wearing off” phenomenon, dyskinesia, residual tremor from insufficient dosage, or a comorbid non-parkinsonian tremor such as functional tremor (33, 34). Anticholinergics have been shown to be effective in reducing tremor severity in a proportion of patients with PD; however, none of our patients reported benefits from biperiden or amantadine at therapeutic doses for tremor exacerbated by levodopa (30). It is unclear whether this lack of response is part of the levodopa-exacerbated tremor or represents general tremor unresponsiveness to anti-parkinsonian drugs. We consider the former to be the most likely explanation, as cases 1 and 4 showed improvement with these medications when not taking levodopa. Other medications proposed to treat tremor in PD, such as clozapine or beta-blockers, were not tested in our patients to improve tremor exacerbated by levodopa. Key observations and hypotheses from this study are summarized in Table 2.

**Table 2:** Key findings and hypotheses for levodopa-exacerbated tremor.

Demographic and clinical features
Frequency: 1.46% of whole cohort (1.55% for patients taking levodopa)
Both sexes affected
Rest tremor exacerbates ≥20 min after levodopa intake
Differential diagnosis
Levodopa underdosing
Dopamine-resistant rest tremor
Peak-dose choreic dyskinesia
Wearing off phenomenon
Comorbid tremor syndrome
Possible pathogenic mechanisms
Shifting basal ganglia oscillations
Dopaminergic hypersensitivity
Noradrenergic hyperactivity
Serotoninergic dysfunction
Therapeutic implications
Patients become reluctant to take levodopa, except for low doses
Amantadine and anticholinergics have no apparent benefit
Low levodopa doses may limit the overall therapeutic benefit

Our study has limitations. First, the study was conducted in a referral center for PD, which may introduce selection bias. Second, we did not perform neurophysiological assessments to measure tremor amplitude in patients with tremor exacerbated by levodopa, and the frequency measured with an application requires further validation. This will be important in further studies to quantify the increase in tremor amplitude caused by levodopa objectively. Moreover, dopamine imaging, such as DaT Scan, can be useful to test whether caudate-predominant dopaminergic preservation can help identify patients at risk for levodopa-exacerbated tremor.

## Conclusion

Clinical exacerbation of tremor following levodopa intake is an uncommon side effect in patients with PD that may strongly limit dosing, hampering improvement in other motor manifestations such as rigidity, bradykinesia, or parkinsonian gait. It is unclear whether this side effect is within the pathophysiology of levodopa-induced dyskinesia or whether it has a distinct pathophysiology. In the meantime, for such patients, if tremor control is necessary, non-dopaminergic drugs (such as low-dose blockers, clozapine, amantadine, or anticholinergics) can be attempted, or adjusting the dose of levodopa by using frequent low doses or extended-release formulations to reduce peak concentrations can be tried; however, tremor exacerbated by levodopa seems poorly responsive to anticholinergics and amantadine.

## Data Availability

The raw data supporting the conclusions of this article will be made available by the authors, without undue reservation.

## Supplementary material

Supplementary video Patient from case 1, 5 h after a single dose of 125 mg of levodopa, showed minimal tremor in the “Off” state. However, at 40 min after 125 mg, there is an increase in tremor amplitude in the upper and lower extremities.
